# Supplementary material for: Cardiovascular Risk Factor Control in 70- to 95-Year-Old Individuals: Cross-Sectional Results from the Population-Based AugUR Study
Source: J Clin Med. 2023 Mar 7;12(6):2102. doi: 10.3390/jcm12062102 (PMC10054695; doi:10.3390/jcm12062102)
Supplement: Supplementary file 1 [file jcm-12-02102-s001.zip › jcm-2194856-supplementary.pdf]

SUPPLEMENTARY MATERIAL

Cardiovascular risk factor control in 70- to 95-year-old individuals: cross-sectional results from the population-based AugUR study

Donhauser et al.

Overview

Supplementary Figure S1	Overview of AugUR study recruitment and restriction to the analysed sample
Supplementary Figure S 2	Distribution of LDL-cholesterol, HbA1c, systolic and diastolic blood pressure stratified by respective medication, sex, and age-group
Supplementary Figure S 3	Characterisation of participants by systolic blood pressure (SBP) and eGFR subgroups
Supplementary Table S 1	Characteristics of participants by medication intake
Supplementary Table S 2	Disparities by sex and age in the proportion of unachieved levels
Supplementary Table S 3	Association of unachieved cardiovascular risk factor control with impaired kidney function
Supplementary Table S 4	Sensitivity analysis for the association of unachieved cardiovascular risk factor control with impaired kidney function – part I
Supplementary Table S 5	Sensitivity analysis for the association of unachieved cardiovascular risk factor control with impaired kidney function – part II

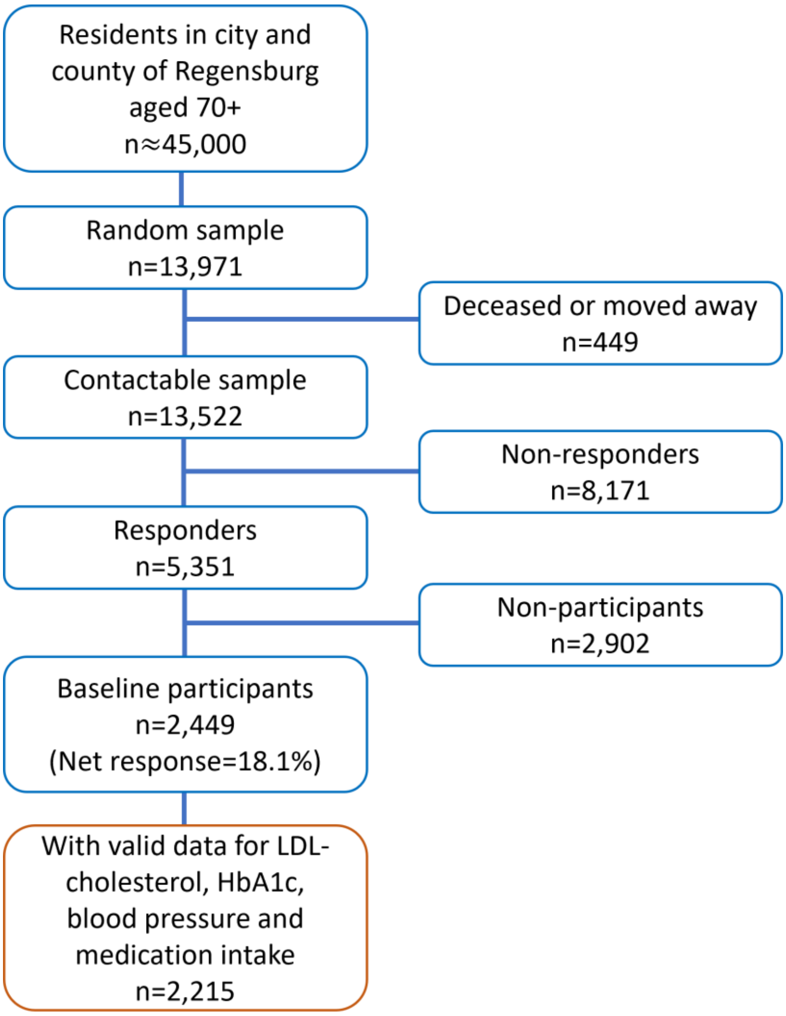

Supplementary Figure S1. Overview of AugUR study recruitment and restriction to the analysed sample.

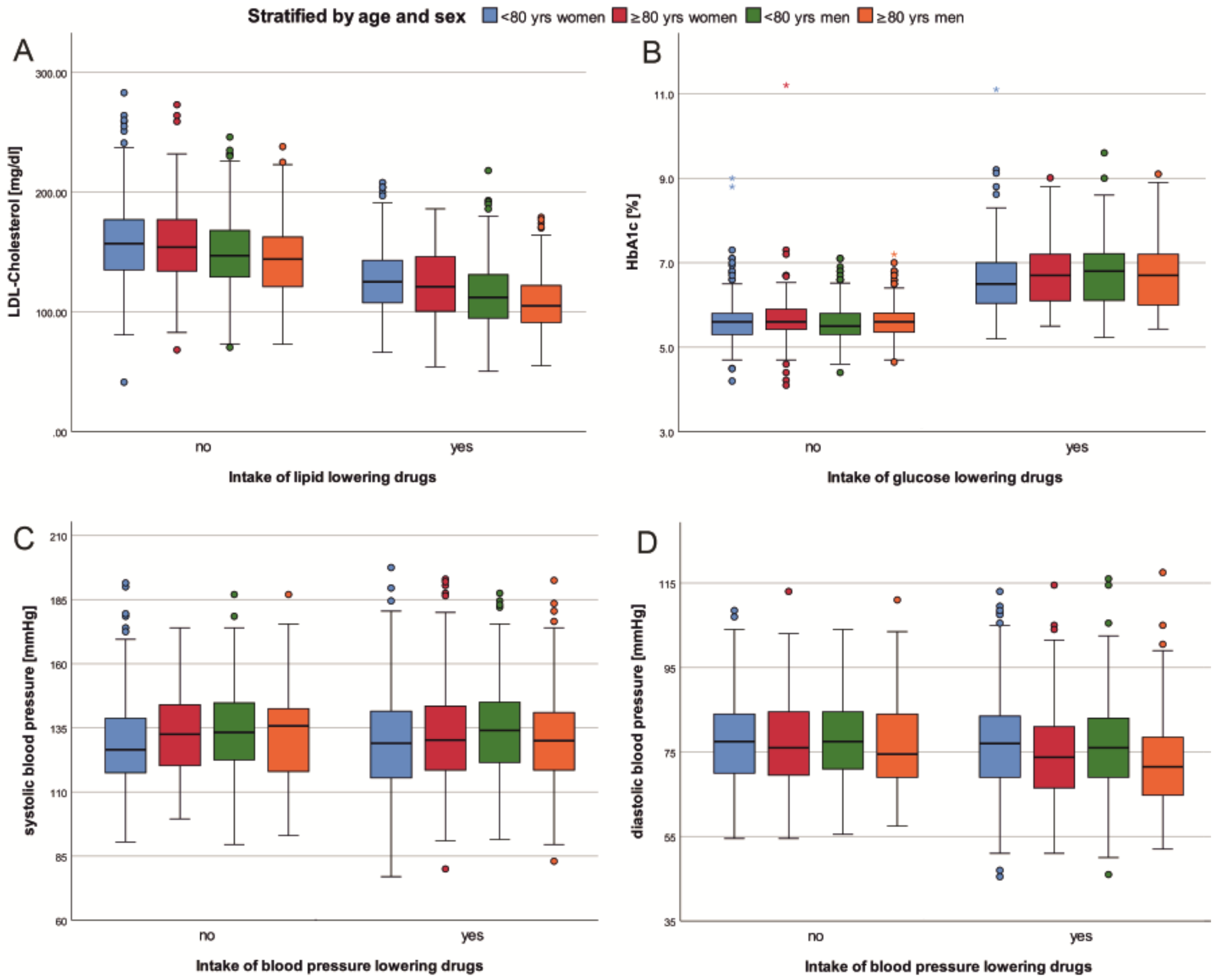

Supplementary Figure S2. Distribution of LDL-cholesterol, HbA1c, systolic and diastolic blood pressure stratified by respective medication, sex, and age-group. Shown are the distribution of LDL-cholesterol stratified by intake of lipid-lowering drugs (A), of HbA1c stratified by intake of glucose-lowering drugs (B), and of systolic as well as diastolic blood pressure stratified by intake of antihypertensive drugs (C and D). Analysed were 2,215 AugUR-participants (n=763 <80 years women, n=401 ≥80 years women, n=706 <80 years men, n=345 ≥80 years men). Shown are median, 25th and 75th percentiles (box), upper and lower whisker (±1.5 IQR), as well as outliers beyond ±1.5 IQR.

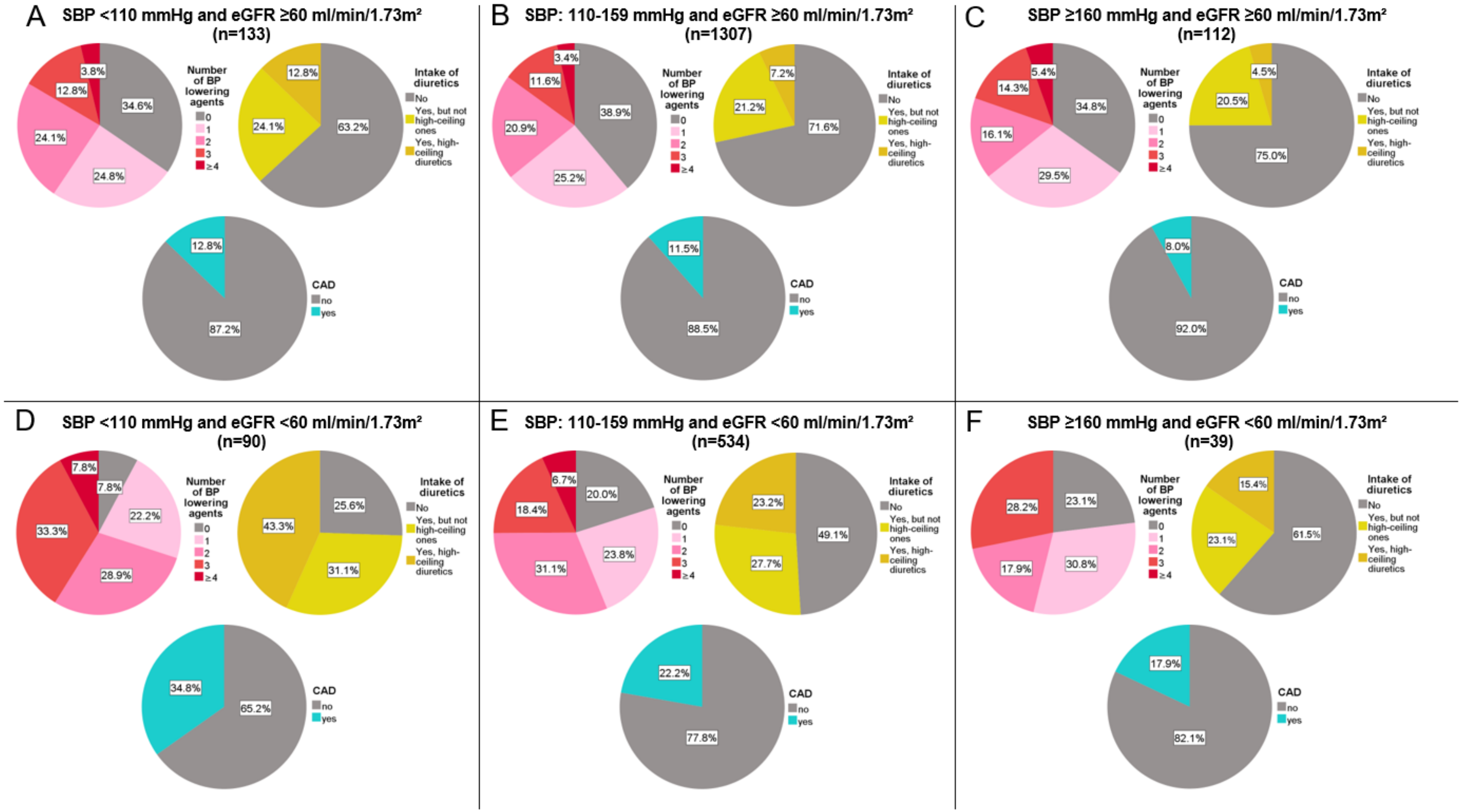

Supplementary Figure S3. Characterisation of participants by systolic blood pressure (SBP) and eGFR subgroups. We characterized participants by SBP with regard to creatinine-based eGFR. Shown are individuals with creatinine-based eGFR ≥60 ml/min/1.73m² (A-C) or <60 ml/min/1.73m² (D-F), each by subgroups of SBP of <110 mmHg (A, D), 110-159 mmHg (B, E), or ≥160 mmHg (C, F). For each panel, we present the proportion of participants with the respective number of different BP lowering agents (red), diuretic intake (yellow), and CAD (blue). Percentages (%) are given compared to the sample size in the respective subgroup.

Supplementary Table S1. Characteristics of participants by medication intake. Characterisation with regard to cardiovascular risk factors and related medical conditions was performed stratified by intake of lipid-, glucose-, blood pressure lowering, and diuretic medication. Shown are numbers and proportions for categorial variables and mean / median as well as standard deviation (SD) / interquartile ratio (IQR) for continuous variables.

	**Intake of lipid-lowering drugs**		**Intake of glucose-lowering drugs**		**Intake of blood pressure lowering drugs**		**Intake of diuretic drugs**	
	**yes (n=772)**	**no (n=1443)**	**yes (n=365)**	**no (n=1850)**	**yes (n=1499)**	**no (n=716)**	**yes (n=802)**	**no (n=1413)**
Men (% (n))	55.6% (429)	43.1% (622)	54.2% (198)	46.1% (853)	47.6% (713)	47.2% (338)	49.5% (397)	46.3% (654)
Age [yrs.] (mean (±SD))	78.4 (±4.9)	78.4 (±5.1)	78.5 (±4.9)	78.3 (±5.1)	78.7 (±5.1)	77.6 (±4.8)	79.2 (±5.2)	77.9 (±4.9)
BMI [kg/m²] (mean (±SD))	28.4 (±4.5)	27.3 (±4.4)	29.9 (±4.9)	27.2 (±4.3)	28.5 (±4.6)	26.0 (±3.8)	29.4 (±4.8)	26.7 (±4.0)
**Cardiovascular Diseases**								
Stroke[a] (% (n))	14.3% (110)	5.6% (81)	12.9% (47)	7.8% (144)	10.3% (154)	5.2% (37)	11.9% (95)	6.8% (96)
CAD[b] (% (n))	32.4% (249)	5.8% (83)	27.0% (98)	12.7% (234)	19.9% (298)	4.8% (34)	24.0% (192)	9.9% (140)
**Lipids**								
LDL-cholesterol [mg/dl] (mean (±SD))	118.4 (±27.7)	153.1 (±32.1)	124.9 (±32.7)	144.2 (±34.3)	137.1 (±35.0)	149.2 (±32.9)	133.9 (±35.8)	145.0 (±33.6)
**Diabetes mellitus**								
Diabetes[c] (% (n))	33.2% (256)	14.9% (215)	100% (365)	5.7% (106)	26.4% (396)	10.5% (75)	32.2% (258)	14.7% (208)
HbA1c [%] (mean (±SD))	5.95 (±0.74)	5.70 (±0.63)	6.73 (±0.88)	5.60 (±0.44)	5.87 (±0.72)	5.62 (±0.54)	5.94 (±0.76)	5.70 (±0.61)
**Blood pressure**								
Hypertension[d] (% (n))	82.8% (639)	67.6% (975)	82.5% (301)	71.0% (1313)	93.3% (1398)	30.2% (216)	92.9% (745)	61.5% (869)
Systolic blood pressure [mmHg] (mean (±SD))	130.4 (±17.5)	132.2 (±18.2)	130.9 (±17.1)	131.7 (±18.1)	131.5 (±18.3)	131.9 (±17.2)	129.5 (±18.6)	132.8 (±17.5)
Diastolic blood pressure [mmHg] (mean (±SD))	74.5 (±10.3)	76.9 (±10.7)	73.9 (±10.4)	76.5 (±10.6)	75.4 (±10.8)	77.6 (±10.2)	73.6 (±10.7)	77.5 (±10.4)
**Kidney function**								
eGFRcrea[e] [ml/min/1.73m²] (mean (±SD))	65.0 (±16.4)	69.2 (±15.8)	62.0 (±18.4)	68.9 (±15.4)	65.2 (±16.7)	73.1 (±13.2)	61.6 (±17.3)	71.2 (±14.3)
eGFRcrea[e] <60 ml/min/1.73m²[h] (% (n))	35.6% (275)	26.9% (388)	43.0% (157)	27.4% (506)	36.0% (540)	17.2% (123)	44.1% (354)	21.9% (309)
eGFRcys[f] [ml/min/1.73m²] (mean (±SD))	59.1 (±16.5)	61.5 (±16.9)	55.1 (±17.8)	61.8 (±16.4)	57.7 (±16.9)	66.8 (±14.8)	52.9 (±16.5)	65.1 (±15.3)
eGFRcys[f] <60 ml/min/1.73m²[h] (% (n))	51.5% (397)	45.0% (646)	60.0% (219)	44.7% (824)	54.2% (809)	32.7% (234)	65.4% (521)	37.0% (522)
UACR[g] [mg/g] (median (IQR))	13.8 (7.4-36.6)	12.6 (6.9-24.7)	17.2 (8.8-51.9)	12.3 (6.8-24.9)	15.0 (8.0-35.6)	10.3 (5.9-18.5)	16.3 (8.5-39.7)	11.8 (6.5-22.3)
UACR[g] ≥30 mg/g (% (n))	21.8% (164)	15.1% (211)	29.3% (103)	15.1% (272)	21.4% (309)	9.4% (66)	22.9% (177)	14.4% (198)

CAD = coronary artery disease; eGFRcrea, eGFRcys = estimated glomerular filtration rate calculated from serum creatinine or serum cystatin C, respectively; UACR = urine albumin-creatinine ratio; [a]) Self-reported history of stroke. 2207 valid values; [b]) self-reported history of myocardial infarction, bypass surgery, and/or stent implantation. 2208 valid values; [c]) self-reported diabetes and/or antidiabetic treatment [1]; [d]) measured blood pressure ≥140/90 mmHg or (antihypertensive treatment and positive self-report) [2]; [e]) via CKD-Epi formula [3]; [f]) via CKD-Epi formula [4]. 2208 valid values; [g]) 2150 valid values.

Supplementary Table S2. Disparities by sex and age in the proportion of unachieved levels. Logistic regression with sex, age-group, its interaction as covariates was performed adjusted for diabetes (if applicable) and CAD/stroke (model I) and additionally for the respective medication intake yes/no (model II). We analysed the 2,204 AugUR-participants with valid information on CAD, stroke, or diabetes. Shown are Odds Ratio (OR) of unachieved levels for men (versus women) and very old age (80+, versus old age 70-79 years), the interaction term (men 80+), corresponding 95% confidence intervals, and p-values.

	**model I**		**model II**	
	**OR (95%CI)**	**p-value**	**OR (95%CI)**	**p-value**
LDL ≥116 mg/dl[a] (n=1650)				
Men vs. women	0.614 (0.473-0.797)	<0.001	0.595 (0.451-0.785)	<0.001
Age ≥80 vs. 70-79 yrs.	0.944 (0.686-1.300)	0.726	0.802 (0.571-1.124)	0.200
Interaction	0.807 (0.526-1.240)	0.329	0.874 (0.554-1.381)	0.565
HbA1c ≥7.0%[b] (n=139)				
Men vs. women	1.690 (1.073-2.663)	0.024	1.475 (0.875-2.487)	0.145
Age ≥80 vs. 70-79 yrs.	1.405 (0.817-2.414)	0.219	1.329 (0.716-2.465)	0.367
Interaction	0.503 (0.240-1.053)	0.068	0.646 (0.275-1.521)	0.317
SBP ≥140 mmHg[c] (n=668)				
Men vs. women	1.363 (1.065-1.744)	0.014	1.373 (1.072-1.758)	0.012
Age ≥80 vs. 70-79 yrs.	1.278 (0.953-1.713)	0.102	1.267 (0.945-1.700)	0.114
Interaction	0.697 (0.456-1.063)	0.094	0.697 (0.456-1.064)	0.094
DBP ≥80 mmHg[d] (n=222)				
Men vs. women	1.048 (0.725-1.514)	0.803	1.050 (0.726-1.517)	0.796
Age ≥80 vs. 70-79 yrs.	1.035 (0.653-1.640)	0.885	1.032 (0.650-1.637)	0.894
Interaction	0.700 (0.340-1.440)	0.333	0.703 (0.341-1.448)	0.339
SBP <120 mmHg[c] (n=568)				
Men vs. women	0.579 (0.444-0.756)	<0.001	0.580 (0.445-0.757)	<0.001
Age ≥80 vs. 70-79 yrs.	0.893 (0.664-1.199)	0.451	0.890 (0.662-1.197)	0.441
Interaction	1.676 (1.079-2.603)	0.021	1.676 (1.079-2.602)	0.021
DBP <80 mmHg[d] (n=1431)				
Men vs. women	0.925 (0.726-1.178)	0.527	0.929 (0.729-1.184)	0.550
Age ≥80 vs. 70-79 yrs.	1.320 (0.985-1.768)	0.063	1.298 (0.968-1.741)	0.081
Interaction	1.135 (0.736-1.749)	0.567	1.141 (0.740-1.760)	0.550

OR = odds ratio; 95%CI = 95% confidence interval; LDL = LDL-cholesterol; SBP = systolic blood pressure; DBP = diastolic blood pressure; [a]) reference is LDL <116 mg/dl (n=554); [b]) reference is HbA1c <7.0% (n=2065); no adjustment for diabetes; [c]) reference is SBP 120-140 mmHg (n=950); [d]) reference is DBP 80-90 mmHg (n=551).

Supplementary Table S3. Association of unachieved cardiovascular risk factor control with impaired kidney function. Shown are Odds Ratios (OR), 95% confidence intervals (CI) and p-values for the association of unachieved risk factor control with creatinine or cystatin-based eGFR <60 ml/min/1.73m² or UACR ≥30 mg/g. We applied logistic regression adjusted for age, sex, CAD/stroke, diabetes (if applicable), obesity, smoking, and respective medication intake (~b0+b1age+b2sex+b3CAD/stroke+b4diabetes+b5obesity+b6smoking+b7treatment+b8control) without interaction (overall model). We also applied a model with interaction between treatment and risk factor control (+b9treatment*control) deriving ORs for treated individuals by exp(b8+b9) and respective 95% CIs. For low SBP/DBP, we used two variables for "treatment" ("treated without diuretics": 1=antihypertensives without diuretics, 0=else; "treated with diuretics": 1=diuretics, 0=else) and respective interaction parameters.

			OR (95%CI)	p-value
Creatinine-based eGFR <60 ml/min/1.73m² [a]	LDL ≥116 mg/dl [d]	Overall	1.093 (0.854-1.398)	$p_{overall}$=0. 479
		Untreated	0.963 (0.663-1.399)	$p_{untreated}$=0.843, $p_{interaction}$=0.382
		Treated	1.197 (0.869-1.648)	
	HbA1c ≥7.0% [e]	Overall	1.422 (0.928-2.180)	$p_{overall}$=0.106
		Untreated	3.075 (1.032-9.165)	$p_{untreated}$=0.044, $p_{interaction}$=0.132
		Treated	1.238 (0.780-1.966)	
	SBP ≥140 mmHg [f]	Overall	0.788 (0.620-1.001)	$p_{overall}$=0.051
		Untreated	0.641 (0.390-1.055)	$p_{untreated}$=0.08, $p_{interaction}$=0.351
		Treated	0.840 (0.639-1.105)	
	DBP ≥90 mmHg [g]	Overall	0.691 (0.459-1.039)	$p_{overall}$=0.076
		Untreated	0.904 (0.437-1.868)	$p_{untreated}$=0.785, $p_{interaction}$=0.389
		Treated	0.614 (0.375-1.006)	
	SBP <120 mmHg [f]	Overall	1.274 (1.005-1.616)	$p_{overall}$=0.045
		Untreated	0.717 (0.428-1.200)	$p_{untreated}$=0.205, $p_{interaction1}$=0.198, $p_{interaction2}$=0.003
		Treated without diuretics	1.115 (0.723-1.721)	
		Treated with diuretics	1.837 (1.294-2.609)	
	DBP <80 mmHg [g]	Overall	1.093 (0.862-1.386)	$p_{overall}$=0.463
		Untreated	0.979 (0.612-1.566)	$p_{untreated}$=0.929, $p_{interaction1}$=0.810, $p_{interaction2}$=0.275
		Treated without diuretics	0.908 (0.608-1.354)	
		Treated with diuretics	1.366 (0.942-1.981)	
Cystatin-based eGFR <60 ml/min/1.73m² [b]	LDL ≥116 mg/dl [d]	Overall	0.804 (0.633-1.022)	$p_{overall}$=0.075
		Untreated	0.703 (0.491-1.006)	$p_{untreated}$=0.054, $p_{interaction}$=0.323
		Treated	0.894 (0.650-1.229)	
	HbA1c ≥7.0% [e]	Overall	1.042 (0.668-1.625)	$p_{overall}$=0.856
		Untreated	1.271 (0.419-3.851)	$p_{untreated}$=0.672, $p_{interaction}$=0.701
		Treated	1.003 (0.618-1.628)	
	SBP ≥140 mmHg [f]	Overall	0.810 (0.646-1.014)	$p_{overall}$=0.066
		Untreated	0.727 (0.476-1.111)	$p_{untreated}$=0.141, $p_{interaction}$=0.557
		Treated	0.845 (0.647-1.103)	
	DBP ≥90 mmHg [g]	Overall	0.899 (0.627-1.289)	$p_{overall}$=0.562
		Untreated	1.115 (0.597-2.084)	$p_{untreated}$=0.733, $p_{interaction}$=0.412
		Treated	0.809 (0.521-1.257)	
	SBP <120 mmHg [f]	Overall	1.509 (1.194-1.906)	$p_{overall}$=0.001
		Untreated	1.422 (0.937-2.158)	$p_{untreated}$=0.098, $p_{interaction1}$=0.94, $p_{interaction2}$=0.538
		Treated without diuretics	1.390 (0.917-2.107)	
		Treated with diuretics	1.698 (1.158-2.488)	
	DBP <80 mmHg [g]	Overall	1.383 (1.104-1.733)	$p_{overall}$=0.005
		Untreated	1.598 (1.059-2.410)	$p_{untreated}$=0.025, $p_{interaction1}$=0.505, $p_{interaction2}$=0.431
		Treated without diuretics	1.321 (0.904-1.932)	
		Treated with diuretics	1.275 (0.867-1.873)	

			OR (95%CI)	p-value
UACR ≥30 mg/g[c]	LDL ≥116 mg/dl[d]	Overall	0.754 (0.570-0.998)	$p_{overall}$=0.049
		Untreated	0.713 (0.468-1.085)	$p_{untreated}$=0.114, $p_{interaction}$=0.725
		Treated	0.787 (0.544-1.139)	
	HbA1c ≥7.0%[e]	Overall	1.248 (0.785-1.982)	$p_{overall}$=0.349
		Untreated	1.388 (0.370-5.211)	$p_{untreated}$=0.627, $p_{interaction}$=0.867
		Treated	1.230 (0.752-2.012)	
	SBP ≥140 mmHg[f]	Overall	1.305 (1.001-1.700)	$p_{overall}$=0.049
		Untreated	1.295 (0.715-2.347)	$p_{untreated}$=0.393, $p_{interaction}$=0.979
		Treated	1.307 (0.972-1.756)	
	DBP ≥90 mmHg[g]	Overall	2.122 (1.406-3.203)	$p_{overall}$<0.001
		Untreated	2.604 (1.193-5.683)	$p_{untreated}$=0.016, $p_{interaction}$=0.546
		Treated	1.964 (1.212-3.183)	
	SBP <120 mmHg[f]	Overall	0.758 (0.556-1.032)	$p_{overall}$=0.079
		Untreated	0.947 (0.459-1.953)	$p_{untreated}$=0.882, $p_{interaction1}$=0.318, $p_{interaction2}$=0.728
		Treated without diuretics	0.594 (0.339-1.042)	
		Treated with diuretics	0.815 (0.529-1.257)	
	DBP <80 mmHg[g]	Overall	0.898 (0.675-1.195)	$p_{overall}$=0.459
		Untreated	0.946 (0.492-1.820)	$p_{untreated}$=0.868, $p_{interaction1}$=0.735, $p_{interaction2}$=0.999
		Treated without diuretics	0.825 (0.523-1.301)	
		Treated with diuretics	0.947 (0.611-1.468)	

OR = odds ratio; 95%CI = 95% confidence interval; eGFR = estimated glomerular filtration rate; UACR = urine albumin-creatinine ratio; LDL = LDL-cholesterol; SBP = systolic blood pressure; DBP = diastolic blood pressure; [a]) serum-creatinine-based eGFR [3]. 2204 valid values; [b]) serum-cystatin-based eGFR [4]. 2197 valid values; [c]) 2140 valid values; [d]) reference is LDL <116 mg/dl; [e]) reference is HbA1c <7.0%.; no adjustment for diabetes; [f]) reference is SBP 120-140 mmHg; [g]) reference is DBP 80-90 mmHg.

Supplementary Table S4. Sensitivity analysis for the association of unachieved cardiovascular risk factor control with impaired kidney function – part I. These are the same analyses as in Supplementary Table 3, but without adjustment for CAD/stroke or diabetes.

			OR (95%CI)	p-value
Creatinine-based eGFR <60 ml/min/$1.73m^2$[a]	LDL ≥116 mg/dl[d]	Overall	0.970 (0.764-1.230)	$p_{overall}$=0.800
		Untreated	0.944 (0.653-1.365)	$p_{untreated}$=0.761, $p_{interaction}$=0.855
		Treated	0.987 (0.726-1.345)	
	HbA1c ≥7.0%[e]	Overall	1.601 (1.051-2.439)	$p_{overall}$=0.028
		Untreated	4.107 (1.377-12.248)	$p_{untreated}$=0.011, $p_{interaction}$=0.068
		Treated	1.362 (0.866-2.143)	
	SBP ≥140 mmHg[f]	Overall	0.772 (0.609-0.978)	$p_{overall}$=0.032
		Untreated	0.622 (0.380-1.017)	$p_{untreated}$=0.059, $p_{interaction}$=0.323
		Treated	0.825 (0.630-1.081)	
	DBP ≥90 mmHg[g]	Overall	0.687 (0.458-1.032)	$p_{overall}$=0.070
		Untreated	0.898 (0.436-1.850)	$p_{untreated}$=0.771, $p_{interaction}$=0.387
		Treated	0.611 (0.374-0.998)	
	SBP <120 mmHg[f]	Overall	1.281 (1.014-1.620)	$p_{overall}$=0.038
		Untreated	0.718 (0.429-1.200)	$p_{untreated}$=0.206, $p_{interaction1}$=0.186, $p_{interaction2}$=0.003
		Treated without diuretics	1.127 (0.734-1.728)	
		Treated with diuretics	1.834 (1.298-2.589)	
	DBP <80 mmHg[g]	Overall	1.148 (0.909-1.450)	$p_{overall}$=0.246
		Untreated	1.005 (0.630-1.602)	$p_{untreated}$=0.984, $p_{interaction1}$=0.995, $p_{interaction2}$=0.280
		Treated without diuretics	1.003 (0.677-1.486)	
		Treated with diuretics	1.393 (0.966-2.006)	
Cystatin-based eGFR <60 ml/min/$1.73m^2$[b]	LDL ≥116 mg/dl[d]	Overall	0.724 (0.575-0.913)	$p_{overall}$=0.006
		Untreated	0.673 (0.473-0.957)	$p_{untreated}$=0.027, $p_{interaction}$=0.586
		Treated	0.765 (0.565-1.038)	
	HbA1c ≥7.0%[e]	Overall	1.181 (0.764-1.824)	$p_{overall}$=0.454
		Untreated	1.852 (0.606-5.654)	$p_{untreated}$=0.279, $p_{interaction}$=0.390
		Treated	1.088 (0.679-1.744)	
	SBP ≥140 mmHg[f]	Overall	0.800 (0.642-0.997)	$p_{overall}$=0.047
		Untreated	0.709 (0.469-1.072)	$p_{untreated}$=0.103, $p_{interaction}$=0.497
		Treated	0.839 (0.646-1.088)	
	DBP ≥90 mmHg[g]	Overall	0.878 (0.616-1.252)	$p_{overall}$=0.473
		Untreated	1.090 (0.590-2.013)	$p_{untreated}$=0.783, $p_{interaction}$=0.401
		Treated	0.789 (0.512-1.219)	
	SBP <120 mmHg[f]	Overall	1.502 (1.194-1.888)	$p_{overall=}$0.001
		Untreated	1.392 (0.921-2.102)	$p_{untreated}$=0.116, $p_{interaction1}$=0.473, $p_{interaction2}$=0.446
		Treated without diuretics	1.368 (0.911-2.054)	
		Treated with diuretics	1.727 (1.187-2.510)	
	DBP <80 mmHg[g]	Overall	1.385 (1.112-1.725)	$p_{overall}$=0.004
		Untreated	1.513 (1.013-2.261)	$p_{untreated}$=0.043, $p_{interaction1}$=0.713, $p_{interaction2}$=0.587
		Treated without diuretics	1.366 (0.942-1.977)	
		Treated with diuretics	1.298 (0.890-1.893)	

			OR (95%CI)	p-value
UACR ≥30 mg/g[c]	LDL ≥116 mg/dl[d]	Overall	0.714 (0.543-0.938)	$p_{overall}$=0.016
		Untreated	0.701 (0.463-1.061)	$p_{untreated}$=0.093, $p_{interaction}$=0.909
		Treated	0.723 (0.505-1.037)	
	HbA1c ≥7.0%[e]	Overall	1.388 (0.879-2.191)	$p_{overall}$=0.159
		Untreated	1.722 (0.465-6.380)	$p_{untreated}$=0.416, $p_{interaction}$=0.733
		Treated	1.350 (0.832-2.190)	
	SBP ≥140 mmHg[f]	Overall	1.273 (0.981-1.652)	$p_{overall}$=0.069
		Untreated	1.273 (0.705-2.297)	$p_{untreated}$=0.424, $p_{interaction}$=0.998
		Treated	1.274 (0.952-1.704)	
	DBP ≥90 mmHg[g]	Overall	1.976 (1.326-2.945)	$p_{overall}$=0.001
		Untreated	2.521 (1.167-5.447)	$p_{untreated}$=0.019, $p_{interaction}$=0.469
		Treated	1.808 (1.135-2.885)	
	SBP <120 mmHg[f]	Overall	0.757 (0.558-1.027)	$p_{overall}$=0.074
		Untreated	0.929 (0.452-1.912)	$p_{untreated}$=0.842, $p_{interaction1}$=0.308, $p_{interaction2}$=0.800
		Treated without diuretics	0.579 (0.332-1.009)	
		Treated with diuretics	0.834 (0.545-1.277)	
	DBP <80 mmHg[g]	Overall	0.918 (0.693-1.215)	$p_{overall}$=0.549
		Untreated	0.953 (0.497-1.826)	$p_{untreated}$=0.885, $p_{interaction1}$=0.808, $p_{interaction2}$=0.997
		Treated without diuretics	0.864 (0.552-1.353)	
		Treated with diuretics	0.954 (0.620-1.467)	

OR = odds ratio; 95%CI = 95% confidence interval; eGFR = estimated glomerular filtration rate; UACR = urine albumin-creatinine ratio; LDL = LDL-cholesterol; SBP = systolic blood pressure; DBP = diastolic blood pressure; [a]) serum-creatinine-based eGFR [3]. 2204 valid values; [b]) serum-cystatin-based eGFR [4]. 2197 valid values; [c]) 2140 valid values; [d]) reference is LDL <116 mg/dl; [e]) reference is HbA1c <7.0%; no adjustment for diabetes; [f]) reference is SBP 120-140 mmHg; [g]) reference is DBP 80-90 mmHg.

Supplementary Table S5. Association of unachieved cardiovascular risk factor control with impaired kidney function – part II. These are the same analyses as in Supplementary Table 3, but without adjusting for obesity (yes/no) or smoking (ever/never).

			OR (95%CI)	p-value
Creatinine-based eGFR <60 ml/min/1.73m^2[a]	LDL ≥116 mg/dl[d]	Overall	1.099 (0.860-1.404)	$p_{overall}$=0.451
		Untreated	0.968 (0.667-1.404)	$p_{untreated}$=0.863, $p_{interaction}$=0.380
		Treated	1.203 (0.874-1.652)	
	HbA1c ≥7.0%[e]	Overall	1.516 (0.993-2.314)	$p_{overall}$=0.054
		Untreated	3.457 (1.166-10.248)	$p_{untreated}$=0.025, $p_{interaction}$=0.106
		Treated	1.313 (0.829-2.068)	
	SBP ≥140 mmHg[f]	Overall	0.785 (0.618-0.996)	$p_{overall}$=0.046
		Untreated	0.638 (0.388-1.047)	$p_{untreated}$=0.075, $p_{interaction}$=0.347
		Treated	0.837 (0.637-1.100)	
	DBP ≥90 mmHg[g]	Overall	0.677 (0.451-1.018)	$p_{overall}$=0.061
		Untreated	0.868 (0.421-1.792)	$p_{untreated}$=0.703, $p_{interaction}$=0.423
		Treated	0.607 (0.371-0.995)	
	SBP <120 mmHg[f]	Overall	1.257 (0.993-1.593)	$p_{overall}$=0.058
		Untreated	0.707 (0.423-1.182)	$p_{untreated}$=0.186, $p_{interaction1}$=0.208, $p_{interaction2}$=0.003
		Treated without diuretics	1.087 (0.706-1.675)	
		Treated with diuretics	1.825 (1.287-2.589)	
	DBP <80 mmHg[g]	Overall	1.072 (0.847-1.358)	$p_{overall}$=0.562
		Untreated	0.959 (0.600-1.533)	$p_{untreated}$=0.862, $p_{interaction1}$=0.807, $p_{interaction2}$=0.271
		Treated without diuretics	0.888 (0.597-1.326)	
		Treated with diuretics	1.340 (0.927-1.939)	
Cystatin-based eGFR <60 ml/min/1.73m^2[b]	LDL ≥116 mg/dl[d]	Overall	0.813 (0.642-1.030)	$p_{overall}$=0.087
		Untreated	0.708 (0.496-1.010)	$p_{untreated}$=0.057, $p_{interaction}$=0.304
		Treated	0.906 (0.662-1.238)	
	HbA1c ≥7.0%[e]	Overall	1.124 (0.726-1.740)	$p_{overall}$=0.599
		Untreated	1.542 (0.510-4.656)	$p_{untreated}$=0.443, $p_{interaction}$=0.541
		Treated	1.060 (0.659-1.704)	
	SBP ≥140 mmHg[f]	Overall	0.795 (0.636-0.993)	$p_{overall}$=0.043
		Untreated	0.717 (0.471-1.090)	$p_{untreated}$=0.119, $p_{interaction}$=0.566
		Treated	0.829 (0.637-1.077)	
	DBP ≥90 mmHg[g]	Overall	0.897 (0.627-1.282)	$p_{overall}$=0.551
		Untreated	1.114 (0.599-2.069)	$p_{untreated}$=0.734, $p_{interaction}$=0.405
		Treated	0.807 (0.522-1.249)	
	SBP <120 mmHg[f]	Overall	1.468 (1.164-1.850)	$p_{overall}$=0.001
		Untreated	1.387 (0.916-2.099)	$p_{untreated}$=0.122, $p_{interaction1}$=0.874, $p_{interaction2}$=0.504
		Treated without diuretics	1.387 (0.876-1.999)	
		Treated with diuretics	1.678 (1.150-2.450)	
	DBP <80 mmHg[g]	Overall	1.354 (1.083-1.692)	$p_{overall}$=0.008
		Untreated	1.543 (1.027-2.319)	$p_{untreated}$=0.037, $p_{interaction1}$=0.524, $p_{interaction2}$=0.491
		Treated without diuretics	1.288 (0.885-1.879)	
		Treated with diuretics	1.268 (0.869-1.856)	

			OR (95%CI)	p-value
UACR ≥30 mg/g[c]	LDL ≥116 mg/dl[d]	Overall	0.766 (0.579-1.012)	$p_{overall}$=0.061
		Untreated	0.719 (0.473-1.093)	$p_{untreated}$=0.123, $p_{interaction}$=0.698
		Treated	0.802 (0.556-1.158)	
	HbA1c ≥7.0%[e]	Overall	1.359 (0.861-2.147)	$p_{overall}$=0.188
		Untreated	1.620 (0.437-6.007)	$p_{untreated}$=0.471, $p_{interaction}$=0.781
		Treated	1.329 (0.818-2.157)	
	SBP ≥140 mmHg[f]	Overall	1.286 (0.988-1.672)	$p_{overall}$=0.061
		Untreated	1.277 (0.706-2.308)	$p_{untreated}$=0.419, $p_{interaction}$=0.980
		Treated	1.288 (0.960-1.727)	
	DBP ≥90 mmHg[g]	Overall	2.052 (1.368-3.078)	$p_{overall}$=0.001
		Untreated	2.491 (1.148-5.407)	$p_{untreated}$=0.021, $p_{interaction}$=0.565
		Treated	1.908 (1.185-3.071)	
	SBP <120 mmHg[f]	Overall	0.749 (0.551-1.019)	$p_{overall}$=0.065
		Untreated	0.923 (0.448-1.900)	$p_{untreated}$=0.827, $p_{interaction1}$=0.327, $p_{interaction2}$=0.766
		Treated without diuretics	0.585 (0.334-1.022)	
		Treated with diuretics	0.812 (0.528-1.248)	
	DBP <80 mmHg[g]	Overall	0.878 (0.661-1.167)	$p_{overall}$=0.370
		Untreated	0.919 (0.479-1.765)	$p_{untreated}$=0.800, $p_{interaction1}$=0.729, $p_{interaction2}$=0.961
		Treated without diuretics	0.799 (0.507-1.259)	
		Treated with diuretics	0.937 (0.606-1.451)	

OR = odds ratio; 95%CI = 95% confidence interval; eGFR = estimated glomerular filtration rate; UACR = urine albumin-creatinine ratio; LDL = LDL-cholesterol; SBP = systolic blood pressure; DBP = diastolic blood pressure; [a]) serum-creatinine-based eGFR [3]. 2204 valid values; [b]) serum-cystatin-based eGFR [4]. 2197 valid values; [c]) 2140 valid values; [d]) reference is LDL <116 mg/dl; [e]) reference is HbA1c <7.0%; no adjustment for diabetes; [f]) reference is SBP 120-140 mmHg; [g]) reference is DBP 80-90 mmHg.

References

[1] Meisinger C, Döring A, Heier M, Thorand B, Löwel H. Type 2 diabetes mellitus in Augsburg - An epidemiological overview. Gesundheitswesen 2005;67. https://doi.org/10.1055/s-2005-858251.

[2] Muli S, Meisinger C, Heier M, Thorand B, Peters A, Amann U. Prevalence, awareness, treatment, and control of hypertension in older people: Results from the population-based KORA-age 1 study. BMC Public Health 2020;20:1–10. https://doi.org/10.1186/s12889-020-09165-8.

[3] Levey AS, Stevens LA, Schmid CH, Zhang Y, Castro AF, Feldman HI, et al. A new equation to estimate glomerular filtration rate. Ann Intern Med 2009;150:604–12. https://doi.org/10.7326/0003-4819-150-9-200905050-00006.

[4] Inker LA, Schmid CH, Tighiouart H, Eckfeldt JH, Feldman HI, Greene T, et al. Estimating Glomerular Filtration Rate from Serum Creatinine and Cystatin C. N Engl J Med 2012;367:20–9. https://doi.org/10.1056/nejmoa1114248.
